# Squamous Cell Carcinoma in South-Eastern Equatorial Rain Forest in Calabar, Nigeria

**Published:** 2009-11-16

**Authors:** M. E. Asuquo, I. A. Ikpeme, E. E. Bassey, G. Ebughe

**Affiliations:** ^a^Department of Surgery, University of Calabar Teaching Hospital, Calabar, Nigeria; ^b^Department of Pathology, University of Calabar Teaching Hospital, Calabar, Nigeria

## Abstract

**Background:** In North America and Europe, 80% of invasive skin cancers are basal cell carcinoma while 20% are squamous cell carcinoma (SCC). In contrast, African studies reveal a preponderance of SCC. Risk factors are grouped into solar and nonsolar. Oculocutaneous albinism (OCA) is a known risk factor for skin cancer in Africans. Their contributions vary with race and geographic region. This study sought to evaluate the pattern, risk factors, and outcome of management of this lesion in our setting. **Method:** All the patients with histologic diagnosis of SCC between January 2006 and December 2007 were prospectively studied as part of the wider study of skin cancers. **Results:** The 19 patients (12 males and 7 females) whose ages ranged between 16 and 70 years (mean = 46.2 years) accounted for 51.4% of skin malignancies. Marjolin's ulcers were recorded in 12 patients (63.2%) while 7 patients (36.8%) were non-Marjolin's including 2 OCA patients. The limb was the commonest site involved (57.9%). The outcomes were poor in some cases because of late presentation after topical treatment. **Conclusion:** Chronic ulcers, inflammation, and albinism were identified predisposing factors. Public health education on prevention, early presentation, and surgical evaluation of chronic ulcers would improve outcome.

In the United States, approximately 80% of invasive skin cancers are basal cell carcinoma (BCC) while 20% are squamous cell carcinoma (SCC), making cutaneous SCC the second most common skin cancer.^1^,^2^ In Africa, several studies reveal a preponderance of SCC.^3^–^5^ Among the blacks in the United States, BCC were almost similar in frequency to SCC.^4^ In New Orleans, La, SCC lesions were about 20% more common than BCC lesions in blacks of the same population.^6^ Well-known risk factors shown to promote the development of cutaneous SCC include solar and nonsolar factors. The later includes immunosuppression, exposure to ionizing radiation, chemical carcinogenesis, and infection with human papillomavirus. The host responses that influence the development of SCC are fair skin and chronic inflammation.^2^ Oculocutaneous albinism (OCA) is a known risk factor for skin cancer with SCC as the commonest dermatological malignancy among African albinos.^7^,^8^ Their contributions appear to vary with race, geographic region, and site of lesion. We present this study in an attempt to evaluate the possible risk factors in our setting and recommend measures for its prevention and improvement of outcome.

## MATERIALS AND METHODS

Patients who presented with histologic diagnosis of skin malignancy seen in the University of Calabar Teaching Hospital between January 2006 and December 2007 were prospectively studied. The 19 patients with SCC formed the basis of this report. Indices evaluated were age, gender, risk factors, clinical presentation, histology, treatment, and outcome. This was compared with our previous retrospective report (January 2000 and December 2004).^7^

## RESULTS

Thirty-seven patients had histologic diagnosis of skin malignancy during the study period and the 19 patients with SCC accounted for 51.4% of skin malignancies. The ages of the 19 patients (12 males and 7 females; M:F = 1.7:1) ranged from 16 to 70 years (mean = 46.2 years).

The 12 patients with Marjolin's ulcer accounted for 63.2% of SCC while 7 patients were non-Marjolin's (36.8%). Two patients (10.5%) in the non-Marjolin's group had OCA while the other 5 were blacks. Table 1 shows the site distribution. The commonest site was the limbs 63.2% (upper 5.3% and lower 57.9%) while the external genitalia/perineum ranked second with 3 patients (15.8%), and the anal/perianal area involved 2 patients (10.5%).

Clinical features suspicious for malignancies were raised with or without everted margins with some fungating lesions. The common isolates were *Staphylococcus aureus*, proteus, and pseudomonas. Histopathologic diagnosis, based on multiple biopsies from the edges of the ulcers, showed SCC. All the patients were exposed to periods of topical treatment with local herbal preparations. No patient was human immunodeficiency virus–seropositive.

In the Marjolin's subset, there were 12 patients (9 males and 3 females). Their ages ranged from 16 to 65 years (mean = 44.1 years). Seven patients suffered traumatic injuries from road traffic accidents that involved the lower limbs, while a male had a puncture wound of the left middle finger. Two lesions were scar cancer and one from chronic osteomyelitis. The average time between injury and diagnosis of Marjolin's ulcer (latency period) was 18.1 years. Six patients were offered amputation because of extensive lesion; the one with the finger lesion had Ray amputation, 3 below knee amputations, while 2 others declined a below-knee amputation and were lost to follow-up. Three patients had wide local excision (WLE) and skin grafting with initial good results during the 9- and 11-month follow-up. The fourth patient offered WLE developed a local recurrence 3 months after skin grafting and was referred for radiotherapy. The patient with chronic osteomyelitis with extensive ulcer offered a below-knee amputation developed an inguinal lymphadenopathy and metastasis to the left lung after 6 months. He commenced adjuvant chemotherapy but was lost to follow-up after a month.

**Figure 1:**
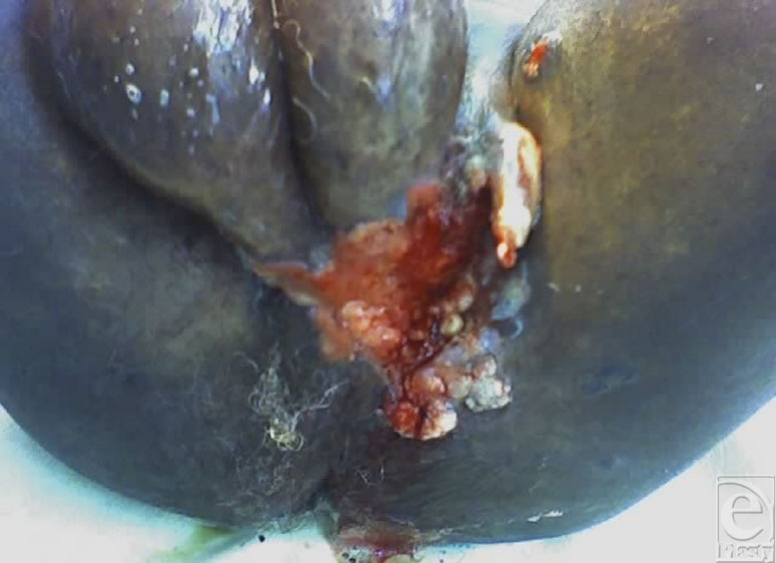
Squamous cell carcinoma.

**Figure 2:**
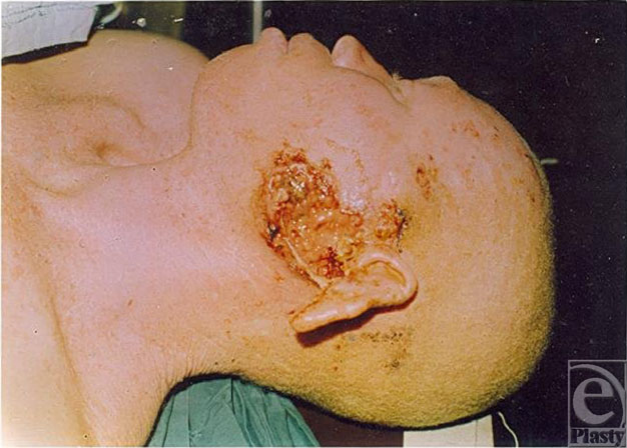
Squamous cell carcinoma in an albino.

The non-Marjolin's subset had 7 patients (4 females and 3 males). The ages of the albinos (a male and a female) were 22 and 27 years, respectively. The ages of the 5 blacks ranged from 50 to 70 years (mean = 60 years). The male patient with a perineal ulcer also had warts. Two patients had advanced anal/perianal lesions, one declined operation while the other who was also incontinent had a terminal colostomy as well as chemotherapy with 5 fluorouracil and levamisole. She was lost to follow-up. Two patients with fungating vulval ulcers had wide excision and skin grafting. They had no evidence of warts, cervical dysplasia, or lichen sclerosus. Both were lost to follow-up. There was a fatal outcome in a patient (Fig 1) with an invasive SCC lesion who developed a recurrence and inguinal lymphadenopathy following WLE. The head region was inflicted in the 2 OCA patients (Fig 2). The outcome was satisfactory in the male after WLE and adjuvant chemotherapy. However, the female with the invasive SCC that inflicted the left infraorbital/orbital region had a poor outcome after excision, adjuvant chemotherapy, and radiotherapy.

## DISCUSSION

Squamous cell carcinoma was the commonest skin malignancy during this study period (51.4%). This is in keeping with our previous report in which 21 SCC cases were reported in 5 years and accounted for 33.3% of skin malignancies.^9^ Yakubu and Mabogunje^5^ reported 524 cases in 12 years at Zaria in northern Nigeria. Other studies in Togo,^3^ Tanzania,^4^ and blacks in America^6^ attest to SCC as the commonest skin malignancy. This contrasts with reports from North America and Europe, where SCC ranked second.^1^,^2^ *The reversal of SCC/BCC, similar to that seen in transplant patients, may be due to oncogenic viruses in the setting of immunosuppression*.^2^ *However, none of our patients had evidence of immunosuppression. This, in the African patients, could well be due to chronic inflammation/malnutrition and possibly parasitic infestation*.^4^,^5^

The lower limb was the commonest site involved. This is in keeping with our earlier report^7^ and other studies.^4^,^5^ Notably, no lesion affected the head and neck region in the blacks, which ranked second in some studies.^4^,^5^ Collectively, the other sites involving perineum, vulva, anus, and perianal regions are non–sun-exposed areas, thus highlighting the role of nonsolar factors in our setting. This is in keeping with other reports from Africa^4^,^5^,^9^,^10^ and blacks in America.^6^ While sun exposure is the major aetiological factor in the whites, chronic ulcer and inflammation appear to be the leading risk factor in the blacks.^4^,^5^,^10^ Chronic leg ulcer was the commonest predisposing factor in Zaria, Nigeria, and most arose from post-burns scar. This was at variance with our report with no case following burn injury. In the Marjolin's subset, chronic trauma–induced ulcer was the predominant risk factor and traumatic injuries from road traffic accidents are largely preventable.

The 2 OCA patients suffered lesions that involved the head region, thus highlighting the role of solar radiation as a risk factor. It should be noted that no black patient suffered any lesion that afflicted the head region in this study. Public education strategy on prevention and early presentation is recommended as albinism is a known risk factor for skin cancer in Africans with SCC as the commonest lesion.^7^,^8^ *However, patients with vitiligo while as pale as albinos have no increased risk of skin cancer, thus highlighting the fundamental difference between them*.

Infection with human papilloma virus presents as warts that may progress to SCC. Our male patient, whose outcome was fatal, had numerous warts. However, there was no evidence of genital warts, cervical dysplasia, or associated lichen sclerosus in the females.

All the patients were exposed to periods of topical treatment using herbal preparations and this accounted for late presentation. The ingredients contained in the herbs and infection may have accounted for the malignant transformation in chronic wounds. This underscores the need for the surgical evaluation of chronic wounds and scars. Theories of carcinogenesis of Marjolin's ulcer vary from the description of chronic irritation to atypia and to carcinoma in the study by Arons et al.^11^ Newer theories included induction of dominant neoplastic cells, radiation, toxic alteration of mitosis, and implantation of epidermal cells into dermis.^12^

The outcome was poor because of late presentation. There was a fatal outcome in a patient who developed a recurrence with an ipsilateral inguinal lymphadenopathy. Regional lymph nodes should be assessed for metastatic spread as regional metastasis occurs in 2% to 6% of cases and correlate with tumor size, differentiation, and outcome.^2^

Surgery is the most frequent approach of treatment. Wide local excision with a margin of at least 2 to 4 cm of normal appearing tissue is the treatment of choice. Amputation is indicated in deep bone involvement.^13^

In conclusion, SCC is the commonest skin cancer in our center. Nonsolar factors, chronic ulcers, and inflammation were notable risk factors. However, in the albinos, solar radiation was the risk factor in keeping with the experience with the whites. The role of topical herbal preparations requires careful evaluation. Public health education on prevention, early presentation, and surgical evaluation of chronic ulcers would improve outcome.

## Figures and Tables

**Table 1:** Site distribution of squamous cell carcinoma

Site	Total (%)
Head and Neck	2 (10.5)
Trunk	…
Limbs	
Upper	1 (5.3)
Lower	11 (57.9)
External genitalia/perineum	3 (15.8)
Anus/perianal	2 (10.5)
Total	19 (100)

## References

[1] Goldman GD (1998). Squamous cell cancer: a practical approach. Semin Cut Med Surg.

[2] Gross ND, Monroe M (2009). Skin cancer: squamous cell carcinoma.

[3] Napokoura G, Pitche P, Tchangai-Walla K, James K, Kpodzo K (1997). Cutaneous cancer in Togo. Bull Cancer.

[4] Amir H, Kwesigabo G, Hirgi K (1992). Comparative study of superficial cancer in Tanzania. East Afr Med J.

[5] Yakubu A, Mabogunje A (1995). Skin cancer in Zaria Nigeria. Trop Doct.

[6] Mora RG, Perniciaro C (1981). Cancer of the skin in black: 1. A review of 163 black patients with cutaneous squamous cell carcinoma. J Am Acad Dermatol.

[7] Yakubu A, Mabogunje OA (1998). Skin cancer in African albinos. Acta Oncol.

[8] Kromberg JG, Castle D, Zwane EM, Jenkins T (1989). Albinism and skin cancer in Southern Africa. Clin Genet.

[9] Asuquo ME, Ugare G, Odio B, Ebughe G (2006). Squamous cell carcinoma of the skin in Calabar. Niger J Surg Sci.

[10] Ochicha O, Edino ST, Mohammed AZ, Umar AB (2004). Dermatological malignancies in Kano, Northern Nigeria: a histopathological review. Ann Afr Med.

[11] Arons MS, Rodin AE, Lynch JB, Lewis SR (1996). Blocker TG Jr. Scar tissue carcinoma II. An experimental study with special reference to burn scar carcinoma. Ann Surg.

[12] Trent JT, Kirshner RS (2003). Wound and malignancy. Adv Skin Wound Care.

[13] Ames FC, Hicky RC (1980). Squamous cell carcinoma of the skin of the extremeties. Int Adv Surg Oncol.

